# Low Prevalence of Conjunctival Infection with *Chlamydia trachomatis* in a Treatment-Naïve Trachoma-Endemic Region of the Solomon Islands

**DOI:** 10.1371/journal.pntd.0004863

**Published:** 2016-09-07

**Authors:** Robert M. R. Butcher, Oliver Sokana, Kelvin Jack, Colin K. Macleod, Michael E. Marks, Eric Kalae, Leslie Sui, Charles Russell, Helena J. Tutill, Rachel J. Williams, Judith Breuer, Rebecca Willis, Richard T. Le Mesurier, David C. W. Mabey, Anthony W. Solomon, Chrissy h. Roberts

**Affiliations:** 1 Clinical Research Department, Faculty of Infectious and Tropical Diseases, London School of Hygiene & Tropical Medicine, London, United Kingdom; 2 Eye Department, Solomon Islands Ministry of Health and Medical Services, Honiara, Solomon Islands; 3 Sightsavers, Haywards Heath, United Kingdom; 4 Hospital for Tropical Diseases, University College London Hospitals, London, United Kingdom; 5 Primary Care Department, Lata Hospital, Lata, Santa Cruz, Solomon Islands; 6 Bellona Rural Health Centre, Bellona, Solomon Islands; 7 Division of Infection & Immunity, Faculty of Medical Sciences, University College London, London, United Kingdom; 8 Task Force for Global Health, Decatur, Georgia, United States of America; 9 Centre for Eye Research Australia, University of Melbourne, Royal Victorian Eye and Ear Hospital, Melbourne, Australia; University of California San Diego School of Medicine, UNITED STATES

## Abstract

**Background:**

Trachoma is endemic in several Pacific Island states. Recent surveys across the Solomon Islands indicated that whilst trachomatous inflammation—follicular (TF) was present at levels warranting intervention, the prevalence of trachomatous trichiasis (TT) was low. We set out to determine the relationship between chlamydial infection and trachoma in this population.

**Methods:**

We conducted a population-based trachoma prevalence survey of 3674 individuals from two Solomon Islands provinces. Participants were examined for clinical signs of trachoma. Conjunctival swabs were collected from all children aged 1–9 years. We tested swabs for *Chlamydia trachomatis* (*Ct*) DNA using droplet digital PCR. Chlamydial DNA from positive swabs was enriched and sequenced for use in phylogenetic analysis.

**Results:**

We observed a moderate prevalence of TF in children aged 1–9 years (n = 296/1135, 26.1%) but low prevalence of trachomatous inflammation—intense (TI) (n = 2/1135, 0.2%) and current *Ct* infection (n = 13/1002, 1.3%) in children aged 1–9 years, and TT in those aged 15+ years (n = 2/2061, 0.1%). Ten of 13 (76.9%) cases of infection were in persons with TF or TI (p = 0.0005). Sequence analysis of the *Ct*-positive samples yielded 5/13 (38%) complete (>95% coverage of reference) genome sequences, and 8/13 complete plasmid sequences. Complete sequences all aligned most closely to ocular serovar reference strains.

**Discussion:**

The low prevalence of TT, TI and *Ct* infection that we observed are incongruent with the high proportion of children exhibiting signs of TF. TF is present at levels that apparently warrant intervention, but the scarcity of other signs of trachoma indicates the phenotype is mild and may not pose a significant public health threat. Our data suggest that, whilst conjunctival *Ct* infection appears to be present in the region, it is present at levels that are unlikely to be the dominant driving force for TF in the population. This could be one reason for the low prevalence of TT observed during the study.

## Author Summary

Trachoma is the most common infectious cause blindness worldwide, and the target of a global elimination initiative. A package of community-wide interventions is recommended to treat trachoma, which aim to reduce transmission of the causative agent *Chlamydia trachomatis*. These interventions require significant, prolonged investment. Clinical observation of follicles on the underside of the eyelid is used to assess requirement for and success of intervention. However, we now know that there are nonchlamydial causes of these follicles, so the observation of this clinical sign may not be specific for chlamydial infection. A recent study showed that infection testing can be cost-effective in low infection prevalence settings, as the money spent on infection testing is more than offset by the savings from avoiding additional rounds of community-wide interventions. We show here that, despite a high prevalence of clinical signs of disease, the estimated prevalence of chlamydial infection is low. The prevalence of the sight-threatening end stage of disease is also low, so we must consider whether the costly community-wide interventions are appropriate in this setting. The use of molecular tools at the population level to guide trachoma policy is still under investigation; this study will contribute to the pool of data required to assess the utility of these tools.

## Introduction

Trachoma, caused by ocular strains of *Chlamydia trachomatis* (*Ct*), is the leading infectious cause of blindness worldwide [1]. Ocular infection with *Ct* is associated with a characteristic follicular conjunctivitis, known as “trachomatous inflammation—follicular” (TF) [2], which can persist for some time after the initiating infection has been cleared. In some individuals, infection can also cause the sign “trachomatous inflammation—intense” (TI). Repeated and prolonged bouts of severe inflammatory disease can lead to trachomatous scarring [3] (TS) which, in some individuals, can eventually cause the eyelashes to turn inwards, producing trachomatous trichiasis (TT), a condition in which the lashes painfully abrade the cornea. In combination with other trachoma-induced changes to the ocular surface, this may lead to corneal opacity (CO) and blindness [2,4]. In 2014, the World Health Organization (WHO) estimated trachoma to be a public health problem in 51 countries, and responsible for approximately 2.2 million cases of visual impairment. Efforts to globally eliminate the disease as a public health problem are promising, with several countries having reported reaching elimination goals [1].

The island states of the Western Pacific Region are made up of several thousand widely dispersed volcanic islands and coral atolls. It has long been suspected that trachoma is endemic in these islands, with reports from the early twentieth century indicating that trachoma was present [5–9]. More recently, trachoma rapid assessments (TRA) conducted in the Pacific indicated the presence of trachoma in Fiji, Vanuatu, Solomon Islands, Nauru and Kiribati. Although TRAs do not give accurate estimates of disease prevalence, the TRA data suggested that, whilst TF levels appeared high, both TI and TT were surprisingly scarce [10].

A population-based prevalence survey (PBPS) is the gold standard for estimating district-wide trachoma burden [11] and trachoma PBPSs took place between 2011 and 2014 in some districts of the Solomon Islands, Kiribati and Fiji [12]. They reported that TF prevalence was above the WHO-recommended threshold (≥10% TF in children aged 1–9 years) for public health interventions in the Melanesian-dominated districts studied (Solomon Islands and Fiji). Those surveys also reported surprisingly low levels of TT [12,13] (at or below the WHO elimination threshold of 0.1% in the all-ages population[14]) compared to those observed in other populations with highly prevalent TF [15,16].

The seemingly discrepant (with respect to out-of-region comparators) finding of high prevalence of TF in populations with negligible levels of TI or TT led us to question whether ocular *Ct* infections are present in Melanesia. We augmented GTMP mapping of Temotu and Rennell and Bellona provinces in the Solomon Islands, with tests for infection and next-generation sequencing, to determine the prevalence of *Ct*, and whether *Ct* and active trachoma were associated. If *Ct* was detected in conjunctival specimens, we considered whether those strains were of an ocular or genital genotype. We considered how our results compare to other published datasets.

## Methods

### Ethics statement

The study adhered to the tenets of the Declaration of Helsinki. The London School of Hygiene & Tropical Medicine Ethics Committee (6319 and 6360) and the Solomon Islands National Health Research and Ethics Committee (HRC13/18) granted ethical approval for this study.

Village and household heads were consulted prior to enrolment. Individuals were informed of the nature and requirements of the study prior to enrolment, by a staff member fluent in local dialects, and were asked to provide written evidence of consent. For those aged 18 years and under, written consent of a parent or guardian was required.

### Study design

Our study was conducted alongside GTMP survey teams as they undertook mapping of one evaluation unit (EU) in the Solomon Islands in October and November, 2013. In the GTMP study, an EU was defined as a single administrative province, however, Temotu and Rennell and Bellona were grouped together into a single EU due to their small populations. Local healthcare workers identified Temotu and Rennell and Bellona as the provinces with the highest suspected trachoma burden in the country. The study was a cross-sectional cluster-randomised PBPS of trachoma. We determined *a priori* that 1019 children aged 1–9 years should be sampled to estimate infection prevalence of 10% with a precision of ±3% at the 95% confidence level, assuming a design effect of 2.65 [17]. Our sample size was framed around the population of children in this age range, as they are the group most likely to harbour infection [15,18].

The Land Registry listed 533 villages across the two provinces in 2013. Local healthcare workers identified villages that were not currently inhabited, and remaining villages were eligible for simple random selection. In each cluster a targeted number of households were randomly selected from a full list of village households to recruit the required number of children. All household residents over the age of 1 year in each selected household were eligible for inclusion [17].

### Clinical examination and photography

Clinical examination was conducted by graders who had been certified according to GTMP protocols [17]. Clinical grading was carried out using the WHO simplified system, in which trichiasis is defined as at least one eyelash in contact with the eyeball (or evidence of recent removal of in-turned eyelashes), TF is defined as 5 or more follicles of >0.5mm diameter on the central part of the upper tarsal conjunctiva, and TI is defined as pronounced inflammatory thickening of the upper tarsal conjunctiva obscuring >50% of the deep tarsal vessels [2].

Conjunctival photographs were taken using a Nikon D3000 SLR camera and graded by an independent photograder who had previously had a kappa agreement score in excess of 0.9 when grading photographs also graded by a master grader. The photograder was masked to the corresponding field grade for each photograph.

### Clinical sample collection, handling and processing

Conjunctival swabs were collected from all children aged 1–9 years. Polyester-coated cotton swabs (Puritan Medical Products, Guilford, ME, USA) were passed three times over the right tarsal conjunctiva with a 120° turn in between each pass [19–21]. The examiner changed their gloves between participants to avoid cross contamination in the field. 1 in 30 swabs did not touch the conjunctiva but were passed within 15 cm of a seated participant then stored and processed in identical fashion to other study specimens, to act as field contamination controls. Swabs were stored immediately in RNAlater (Life Technologies, Paisley, UK), kept on ice packs in the field before short-term storage at 4°C, and frozen within 48 hours of collection [22]. Specimens were transported to the UK on dry ice where they were stored at -80°C until they were extracted with Qiagen AllPrep DNA/RNA mini kits (Qiagen, Manchester, UK) according to manufacturer’s recommendations.

### Droplet digital PCR

DNA specimens were tested for the *Ct* plasmid using a droplet digital PCR assay targeting a single *Ct* plasmid target in duplex with a human ribonuclease gene, which acted as endogenous control. The published assay methodology was used [23] with minor protocol adjustments to the tested sample volume (4.95μL increased to 8μL) and oligonucleotide concentrations (primer concentration increased from 300nM to 900nM; probe concentration decreased from 300nM to 200nM). Samples were considered valid if there was >95% confidence in non-zero endogenous control concentration, and positive according to published criteria (>95% confidence in non-zero chlamydial plasmid load [23]). Samples from children with TF were retested with an alternative, quantitative assay targeting both chlamydial chromosomal and plasmid targets [24]. The oligonucleotides used are shown in Table 1.

**Table 1:** Oligonucleotides used in this study. Chlamydial targets adapted for ddPCR [23,24] based on sequences from Pickett and colleagues [87]. Endogenous control target adapted from Luo and colleagues [88].

Target	Oligo	Sequence (5ʹ to 3ʹ)
*Chlamydia trachomatis omcB*	Primer (F)	GACACCAAAGCGAAAGACAACAC
	Primer (R)	ACTCATGAACCGGAGCAACCT
	Probe	FAM**-CCACAGCAAAGAGACTCCCGTAGACCG-BHQ1
*Chlamydia trachomatis* plasmid ORF2	Primer (F)	CAGCTTGTAGTCCTGCTTGAGAGA
	Primer (R)	CAAGAGTACATCGGTCAACGAAGA
	Probe	FAM*/HEX**-CCCCACCATTTTTCCGGAGCGA-BHQ1
*Homo sapiens RPP30*	Primer (F)	AGATTTGGACCTGCGAGCG
	Primer (R)	GAGCGGCTGTCTCCACAAGT
	Probe	HEX*-TTCTGACCTGAAGGCTCTGCGCG-BHQ1

F: forward; R: reverse; BHQ: Black Hole Quencher; omcB: outer membrane protein complex B; ORF: open reading frame; RPP30: ribonuclease P/MRP 30kDa subunit

*Diagnostic assay [23]

**Quantitative assay [24]

### Chlamydial genome and plasmid sequencing

Chlamydial DNA was preferentially enriched in clinical samples using custom *Ct*-specific RNA baits in a SureSelect system, as developed by the PATHSEEK consortium. Due to the low biomass of conjunctival swabs, human carrier DNA was added to achieve the required DNA input concentration for sequencing. Samples were sequenced on the Illumina MiSeq platform [25].

### Literature search

Articles were identified through two specific literature searches to illustrate how our data compared to existing published studies. PubMed hits, references contained within those articles and relevant articles from the authors’ own archives were considered eligible for inclusion. Using the terms “population-based” and “trachoma”, we identified population-level studies where TF had been reported in children aged 1–9 years and TT had been reported in adults over the age of 15 years in the same population. District-level data were extracted where more than a single district was reported on in a single publication. Using the search terms “trachoma” and “infection”, we identified studies reporting population-level nucleic acid-based infection data alongside clinical data on TF (with or without TI). Data from communities who had received one or more rounds of MDA were excluded. During both literature searches, studies where age-specific trachoma prevalence in 1- or 2-year age bands was presented were also reviewed.

### Data recording and analysis

Field data were recorded using a purpose-built Open Data Kit (https://opendatakit.org/) app [17]. Age adjustments were carried out using 5-year census age bands [26]. All analyses were carried out using R version 3.2.2 [27]. The overall agreement between field exam and photographic grade was determined using kappa agreement scores. The relationship between TF and *Ct* infection was tested using logistic regression. *Ct* loads in children with and without TF were compared using the Mann Whitney U test.

Following preliminary assessment of mapping quality to various *Ct* strains, 251-bp paired end reads were trimmed and mapped to *Ct* reference strain AHAR-13 genome sequence (GenBank accession CP000051.1) and B/Jali20 plasmid genome sequence (FM865436.1) using Bowtie 2 [28]. SAMtools and BCFtools were used to index and assemble reads, and bases were called by collapsing reads vertically [29]. Trimmed reads were also mapped to E/Bour (genome HE601870.1, plasmid HE603212.1) to determine whether the choice of reference influenced branching points in the phylogram.

Consensus sequences were submitted for megablast search on National Centre for Biotechnology Information (NCBI) GenBank to determine nearest relatives based on genetic sequence. Whole genomes were aligned with progressiveMauve [30]. A core alignment was generated by extraction and amalgamation of locally collinear blocks using stripSubsetLCBs [31]. Distance matrices and bootstrapped phylogeny was inferred using phangorn, ape and SeqinR packages in R [32–34]. Regions orthologous to *ompA*, *trpA* and the plasticity zone (PZ; a ~20 kb region of the *Ct* chromosome between *dsbB* and *ycfV* [35]) were analysed in isolation due to their disproportionately high variability and influence on pathogenicity compared to the rest of the *Ct* chromosome. These were extracted from consensus sequences using BLASTn in the NCBI BLAST+ suite, and aligned using MUSCLE alignment software [36].

## Results

### Enrolment and disease prevalence

The combined population of Temotu (21,362), and Rennell and Bellona (3041) comprises 4.7% (24,403/515,870) of the total population of the Solomon Islands, according to the 2009 national census [26]. We surveyed 959 households in 32 clusters throughout this EU. 4049 people were enumerated, and 3674 (91%) consented to participate (a total of 17% of the provincial population, average 4.2 people per household). Data was not collected on the reasons people did not take part. The examined population included 1135 children aged 1–9 years (53% male), and 2061 adults aged 15 years and over (42% male). The median age was 18 years (min 1, max 100, inter-quartile range [IQR]: 8–38 years). In this population there were 397 (10.9%) cases of TF, 5 (0.1%) cases of TI, and 2 (0.1%) cases of trichiasis in either eye of subjects of all ages identified by field grading. 84% of cases of TF were bilateral.

In children aged 1–9 years, the prevalence of active trachoma (defined as presence of TF and/or TI in either eye) was 26.3% (TF: 26.1% [296/1135]; TI: 0.2% [2/1135]). The proportion of males in this age group with active trachoma was significantly higher than that of females in the same age group (28.9% [176/608] versus 23.1% [122/527], p = 0.027). When adjusted for age and sex, the prevalence of active trachoma was 22% (95% confidence interval [CI]: 18.5–26.0%). In adults aged 15 years and over, the prevalence of active trachoma was 1.2% (TF: 1% [21/2061]; TI: 0.1% [3/2061]). The prevalence of trichiasis in adults was 0.1% (2/2061). When adjusted for age and sex, the prevalence of TT was 0.04% (95% CI: 0–0.3%). The field team did not recall one case of trichiasis and the other was documented as mild, with a single lash contacting the globe away from the cornea.

Photographs were taken from 3110/3674 (85%) of participants. Preliminary quality control yielded 2418 (78%) photographs that were unsuitable for grading due to quality issues. A total of 692 photographs from study participants of all ages (238 in children aged 1–9 years) were evaluated by an independent grader. Photo-grading according to the simplified grading system agreed with TF in 94% of cases leading to Fleiss’ kappa agreement scores of 0.88, which indicated excellent agreement between photo grader and field grading in TF calls. Cases of TS were noticed in the photo set, although the evidence was insufficient to determine the population prevalence of this sign.

**Fig 1:**
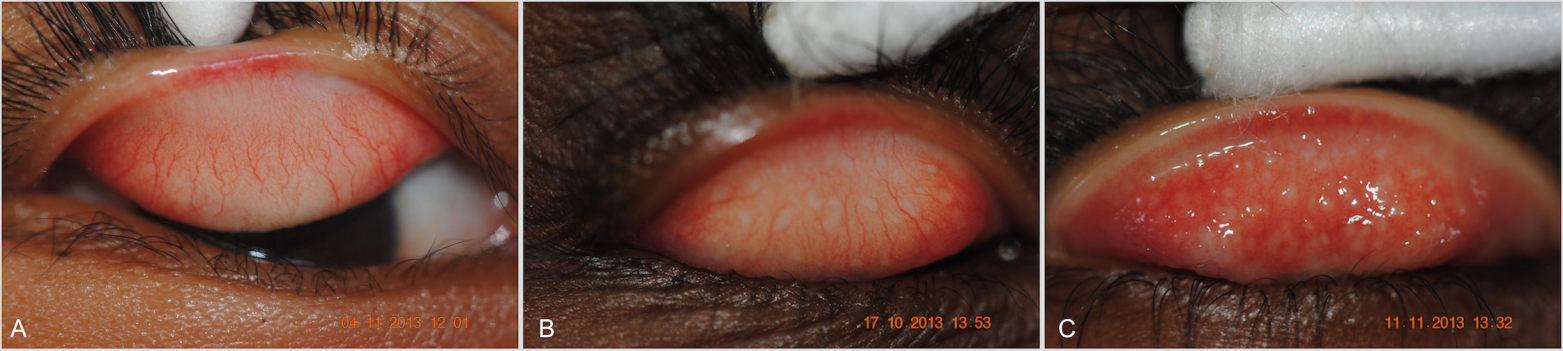
Photographs of conjunctivae showing (**A**) no evidence of active trachoma, (**B**) mild trachomatous inflammation-follicular (TF) and (**C**) more severe TF. All three photographs were taken of conjunctival *C*. *trachomatis* infection-negative children aged 1–9 resident in Temotu, Rennell or Bellona, Solomon Islands, October-November 2013, in whom the photo grade and field grade matched.

**Fig 2:**
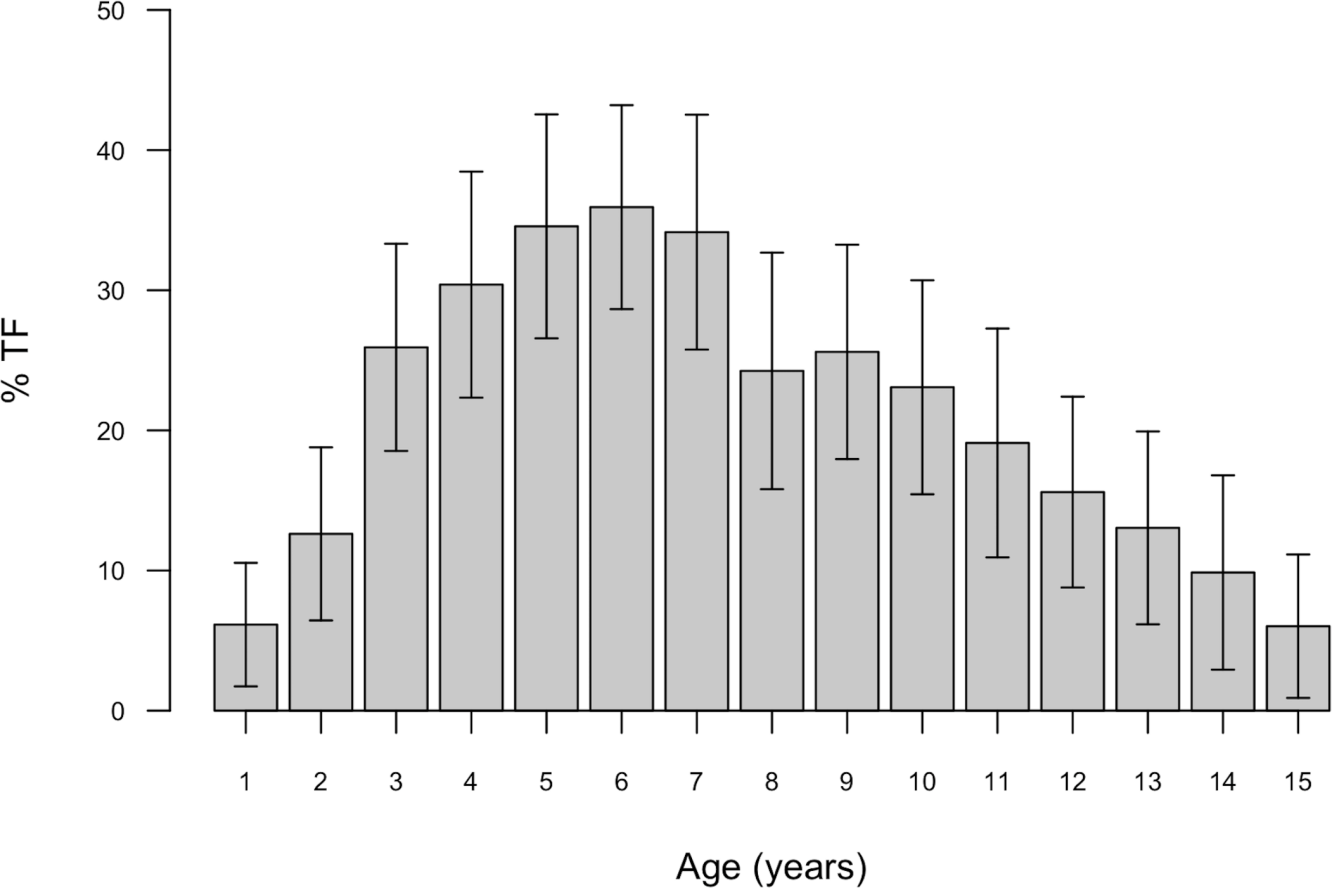
Age-specific prevalence (grey bars) and 95% confidence interval (arrows) of trachomatous inflammation—follicular (TF) in individuals aged 1–15 years, recorded during a trachoma survey of Temotu, Rennell and Bellona, Solomon Islands, October-November 2013.

Fig 1 shows exemplars of both mild and more severe TF in Solomon Island children, as well as a normal conjunctiva for contrast. The age-specific prevalence of TF cases is shown in Fig 2.

### Conjunctival infection

Swabs were collected from 1076/1135 (94.8%) child participants aged 1–9 years, along with 41 field controls. Of those, 1002 (93.1%) passed quality control by testing positive for the endogenous control gene *H*. *sapiens RPP30*. All blank field controls and all known-negative extraction and PCR controls tested negative for endogenous control and microbial targets. Evidence of *Ct* infection was found in 13 (1.3%) of 1002 specimens. Of those who tested positive for plasmid on diagnostic screen, 9 also tested positive for *Ct* chromosomal target *omcB*.

Of the individuals whose swab was positive for the endogenous control *RPP30*, 257/1002 (25.7%) had TF and/or TI in the right eye (Table 2). The prevalence of *Ct* infection was 10/257 (3.9%) in those with TF and/or TI, and 3/745 (0.4%) in those without. Active disease status was highly significantly associated with current infection (odds ratio: 10.0, p = 0.0005).

**Table 2:** Frequency of conjunctival *C*. *trachomatis* infection and active trachoma during a trachoma survey in Temotu, Rennell and Bellona, Solomon Islands, October-November 2013.

ddPCR result	No TF/TI (%)	TF/TI (%)	*Total*
Positive	3 (0.4)	10 (3.9)	**13 (1.3)**
Negative	742 (99.6)	247 (96.1)	**989 (98.7)**
**Total**	**745**	**257**	**1002 (100)**

ddPCR: droplet digital polymerase chain reaction; TF: trachomatous inflammation—follicular; TI: trachomatous inflammation—intense.

While TF was observed in all 32 villages that were surveyed, we observed *Ct* infection in just eight villages. While the study was not designed to detect sub-EU-level differences in prevalence, *post hoc* analysis indicated there were significantly more cases of infection per capita in Rennell and Bellona than in Temotu province (6/131 [4.6%] versus 7/871 [0.8%], respectively; Mann Whitney U: p = 0.0004). TF levels in the 1–9 year old indicator group were in excess of 10% in both provinces included in the EU, but significantly lower in Rennell and Bellona (27.3% in Temotu versus 17.2% in Rennell and Bellona; Mann Whitney U: p = 0.0001).

### Load of infection

The mean load of endogenous control target was 12,560 copies/swab (IQR: 872–13,980 copies/swab). In positive swabs, the median load of *Ct* plasmid was 13,840 copies/swab (IQR: 3599–84,990 copies/swab). There was a large difference in median load between *Ct* positive samples from children with active disease when compared to those without active disease, although the difference was not statistically significant (median 13,840 versus 782 copies/swab; Mann Whitney U test p = 0.81). The median *omcB* load was 7725 copies/swab (IQR: 1696–22,110 copies/swab) and the mean plasmid:genome ratio (i.e., plasmid copy number per bacterium) was 4.4 (IQR: 3.7–5.6) which is similar to that described elsewhere [24].

### Genome sequencing

Sequencing was successful in 11/13 strains. The mean number of paired reads per specimen was 2.3 million (IQR: 2.1–2.7 million; Table 3). The median percentage of reads mapping to A/HAR-13 reference genome was 10.1% (IQR: 1.5–24.4%) per specimen. The median percentage of reads mapping to B/Jali20/OT reference plasmid was 2.1% (IQR: 0.3–4.8%) per specimen.

**Table 3:** Sequencing parameters and results of BLASTn analysis of consensus sequence. Conjunctival *C*. *trachomatis* genomes obtained from Temotu, Rennell and Bellona, Solomon Islands, in October-November 2013.

Sample	Genetic component	Total paired reads	Number of paired reads mapped to reference (%)	% reference covered*	Serovar of closest NCBI BLASTn match
SB000209	Genome	1,948,968	445 (0.02)	0.2	F
	Plasmid		173 (0.01)	15.5	D
SB002563	Genome	1,838,502	513 (< 0.01)	0.5	I
	Plasmid		35 (< 0.01)	12.4	F
SB002739	Genome	2,530,443	403,672 (16.0)	95.8	A
	Plasmid		61,345 (2.4)	99.7	B
SB006908	Genome	2,594,512	34,663 (1.3)	18.5	J
	Plasmid		3857 (0.1)	76.7	D
SB006930	Genome	2,630,055	1,743,718 (66.3)	98.9	A
	Plasmid		293,102 (11.1)	99.8	B
SB008107	Genome	2,269,697	485,840 (21.4)	98.0	A
	Plasmid		79,314 (3.5)	99.8	B
SB011363	Genome	3,878,711	75,326 (1.9)	16.3	I
	Plasmid		17,375 (0.4)	99.2	B
SB011759	Genome	3,044,398	52,165 (1.7)	16.3	A
	Plasmid		11,707 (0.4)	96.0	B
SB011836	Genome	313	0 (0)	0.0	-
	Plasmid		0 (0)	0.0	-
SB012441	Genome	2,996,613	301,482 (10.1)	84.5	A
	Plasmid		63,827 (2.1)	99.5	B
SB013112	Genome	2,274,168	1,345,864 (59.2)	98.9	A
	Plasmid		332,462 (14.6)	99.8	B
SB013321	Genome	2,128,740	584,834 (27.5)	98.8	A
	Plasmid		127,976 (6.0)	98.8	B

* At least 1× read depth

Complete genome sequences (>95% coverage) were obtained from five of 13 specimens, whilst partial genome sequences (<95% coverage) were obtained from six specimens. Complete (>95%) or partial (<95%) plasmid sequences were obtained from eight and three specimens, respectively. One specimen failed to sequence. The median coverage of at least 1× read depth of the reference genome was 51.5% (IQR: 12.4–98.2%); the median coverage of at least 1× of the reference plasmid was 99% (IQR: 61.4–99.7%).

BLASTn analysis of five complete Solomon Islands *Ct* consensus genome sequences against archived *Ct* genome sequences revealed that all five had closest sequence homology and alignment to serovar A type ocular strains. BLASTn analysis of eight complete Solomon Islands plasmid consensus sequences revealed highest sequence homology with published serovar B ocular strain plasmids.

**Fig 3:**
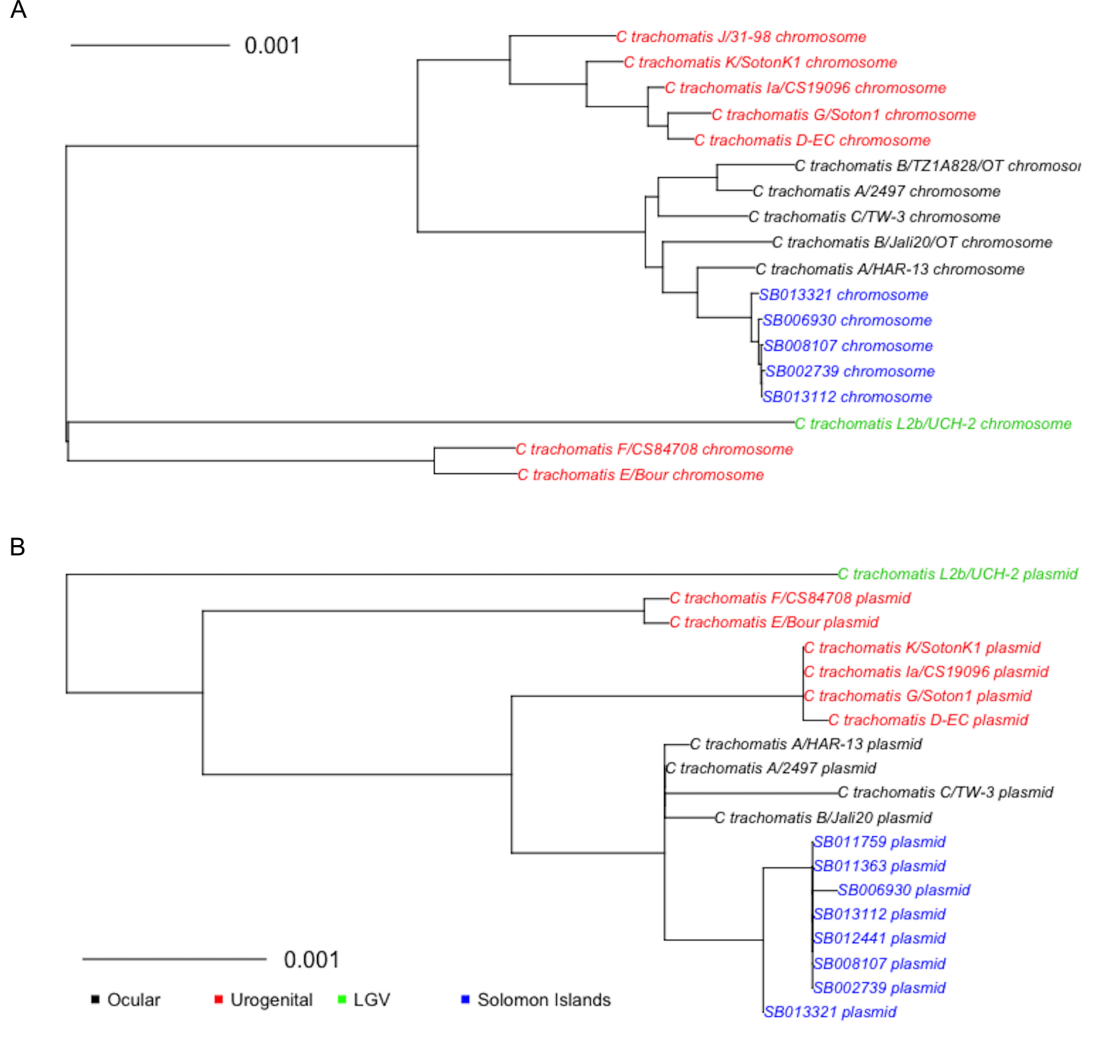
Maximum likelihood phylogram of (**A**) genome and (**B**) plasmid sequences from clinical specimens collected in the Solomon Islands in October and November 2013, assembled to *C*. *trachomatis* A/HAR-13 and B/Jali20/OT reference, respectively. All branches had bootstrap values over 85/100.

Phylogenetic analysis of the complete Solomon Islands *Ct* genomes showed that these samples formed a single clade of closely related genotypes that formed a sub-clade of the ocular strains. The five complete genomes were more closely related to each other than they were to the nearest reference neighbour (A/HAR-13). Strains from the Solomon Islands were most closely related to the ocular serovars in the T2 chlamydial clade, as shown in Fig 3. Bootstrapping supported our phylogram by indicating resampling did not alter the branch position in >80% of bootstrap runs.

Outer membrane protein A (*ompA*) sequences from the ocular reference strains do not cluster in a single clade, as has been described previously [37]. *OmpA* orthologs from the five Solomon Island sequences were more closely related to each other than to their nearest neighbour which was the C-TW3 strain. Their relationship to other reference sequences is shown in S2A Fig. Additionally, tryptophan synthase alpha subunit (*trpA*) orthologs in Solomon Island sequences were most similar to ocular strains (S2B Fig) and featured a single nucleotide deletion leading to a premature stop codon and truncation of the open reading frame when compared to urogenital sequences. Finally, the relationship between the plasticity zone (PZ) of Solomon Island sequences compared to references was evaluated and is shown in supplementary Fig 2C.

### Literature search

**Fig 4:**
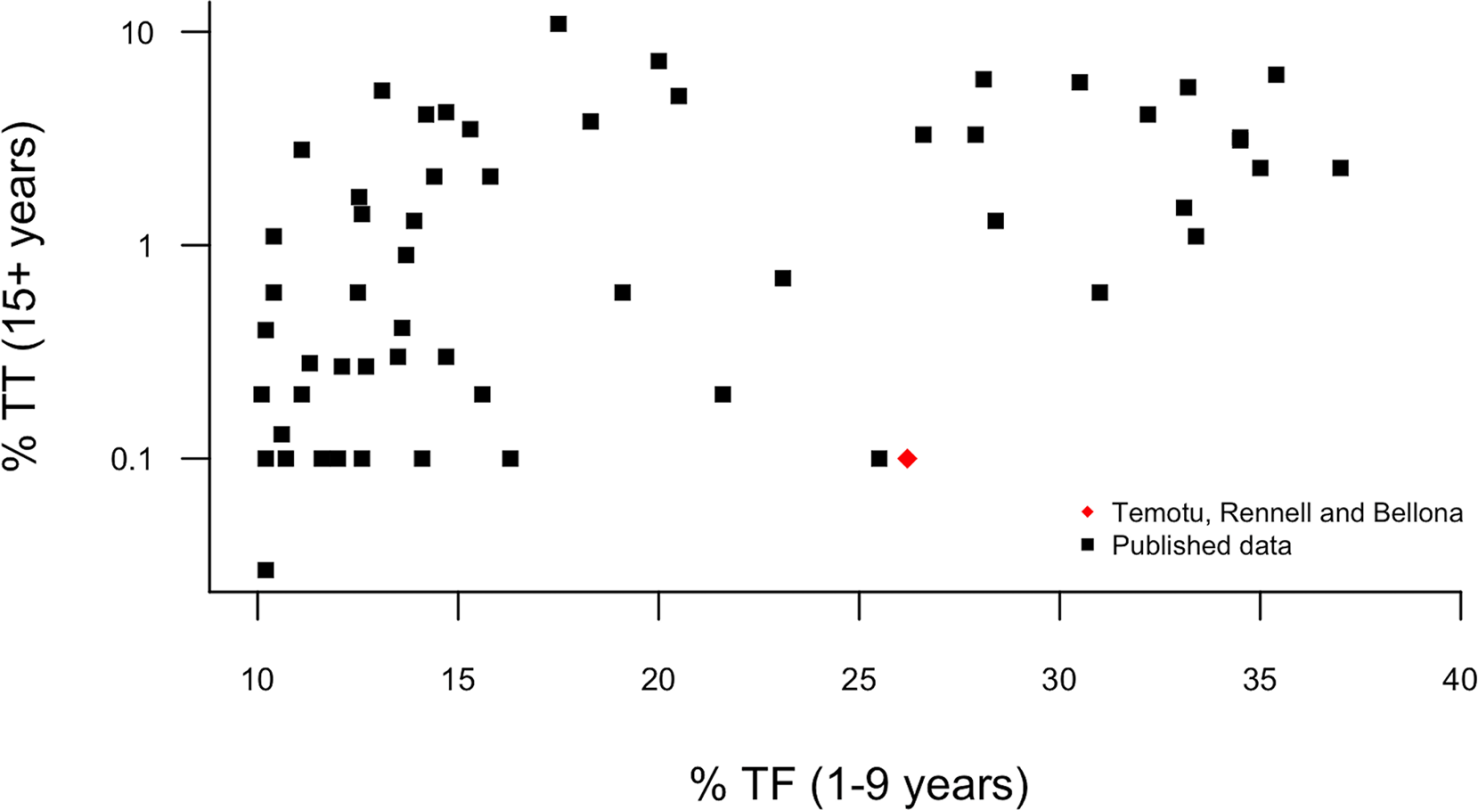
Comparison of unadjusted prevalence of trachomatous trichiasis (TT) in 15+ year olds, and trachomatous inflammation—follicular (TF) in 1–9 year-olds, in treatment-naïve trachoma-endemic EUs for which data have previously been published (n = 58), and in which the TF prevalence in 1–9 year-olds was 10–40%. Correlation coefficient (R) is 0.40 for this subset, but 0.77 if including studies from areas of any prevalence.

A low prevalence of TT has been observed in other populations in which TF was highly prevalent. Fig 4 shows data taken from published PBPSs in Nigeria [38–44], Niger [45], Sudan [46], Kenya [16], Ethiopia [47] and Cameroon [48,49] comparing the prevalence of TF in those aged 1–9 years with the prevalence of TT in 15+ year-olds in the same EUs, all of which were treatment-naïve. Of 58 identified EUs with comparable prevalence of TF in the 1–9 year olds (10% < TF < 40%) to that observed in the current study, 50% had a TT prevalence greater than 1% in those over the age of 15 years; the median TT prevalence was 1%, compared to 0.1% in our survey. Our population had a high TF prevalence when compared to other districts in this analysis with <1% TT.

**Fig 5:**
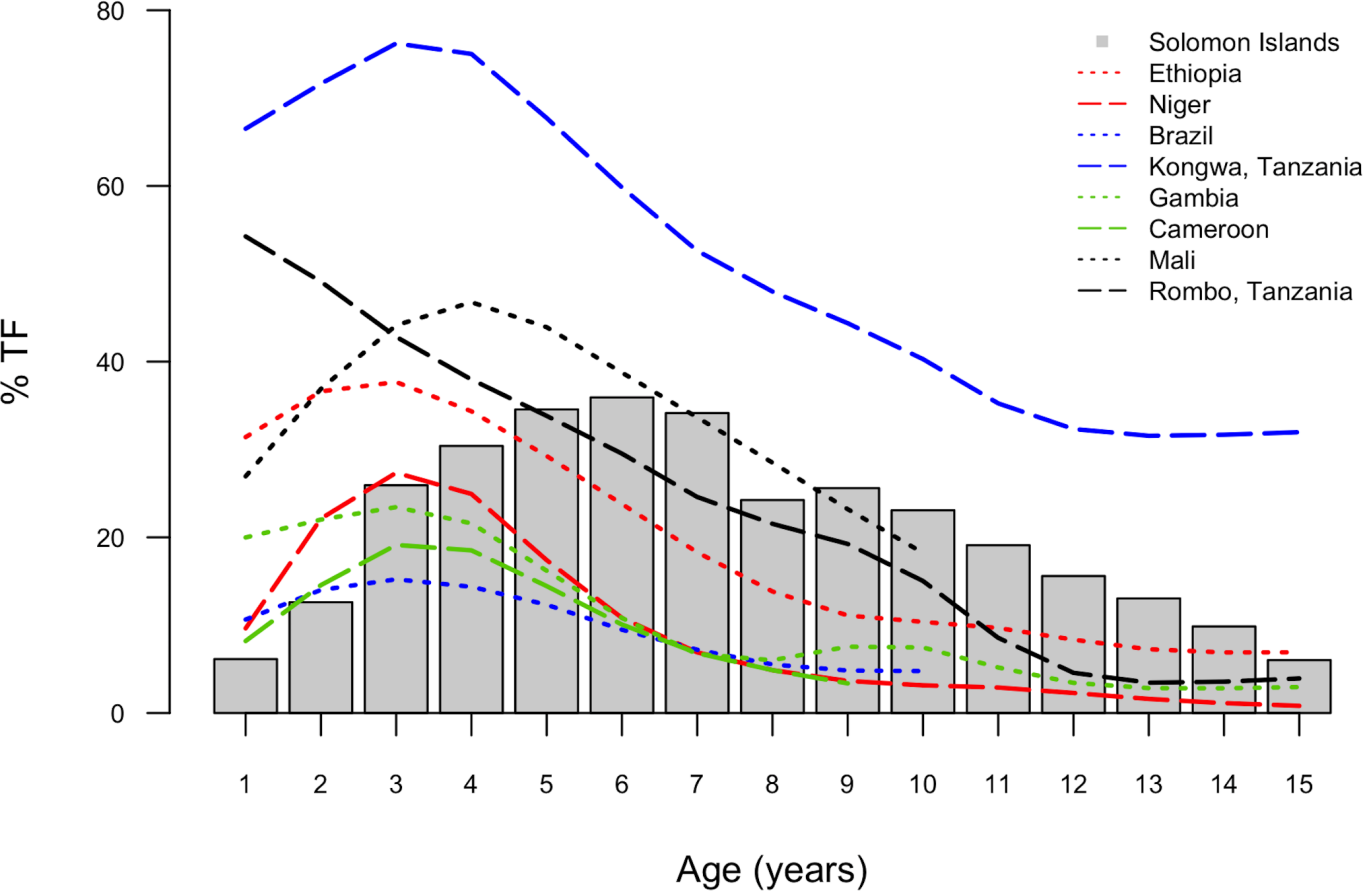
Published age-specific trachomatous inflammation—follicular (TF) prevalence data in studies undertaken in districts with >10% TF in overall child population (dotted and dashed lines), compared to the same in Temotu, Rennell and Bellona, Solomon Islands, October-November 2013 (grey columns).

Published data from studies in Ethiopia [50], Niger [50], Tanzania [18], Gambia [18], Cameroon [48], Mali [51] and Brazil [52] indicate that in other trachoma-endemic populations, the highest age-specific TF prevalence is generally in those aged 3–4 years. When published age-specific TF profiles from other parts of the world are compared to the age distribution in this study (Fig 5), the peak age-specific TF prevalence in our data is shown to be in an older age group.

**Fig 6:**
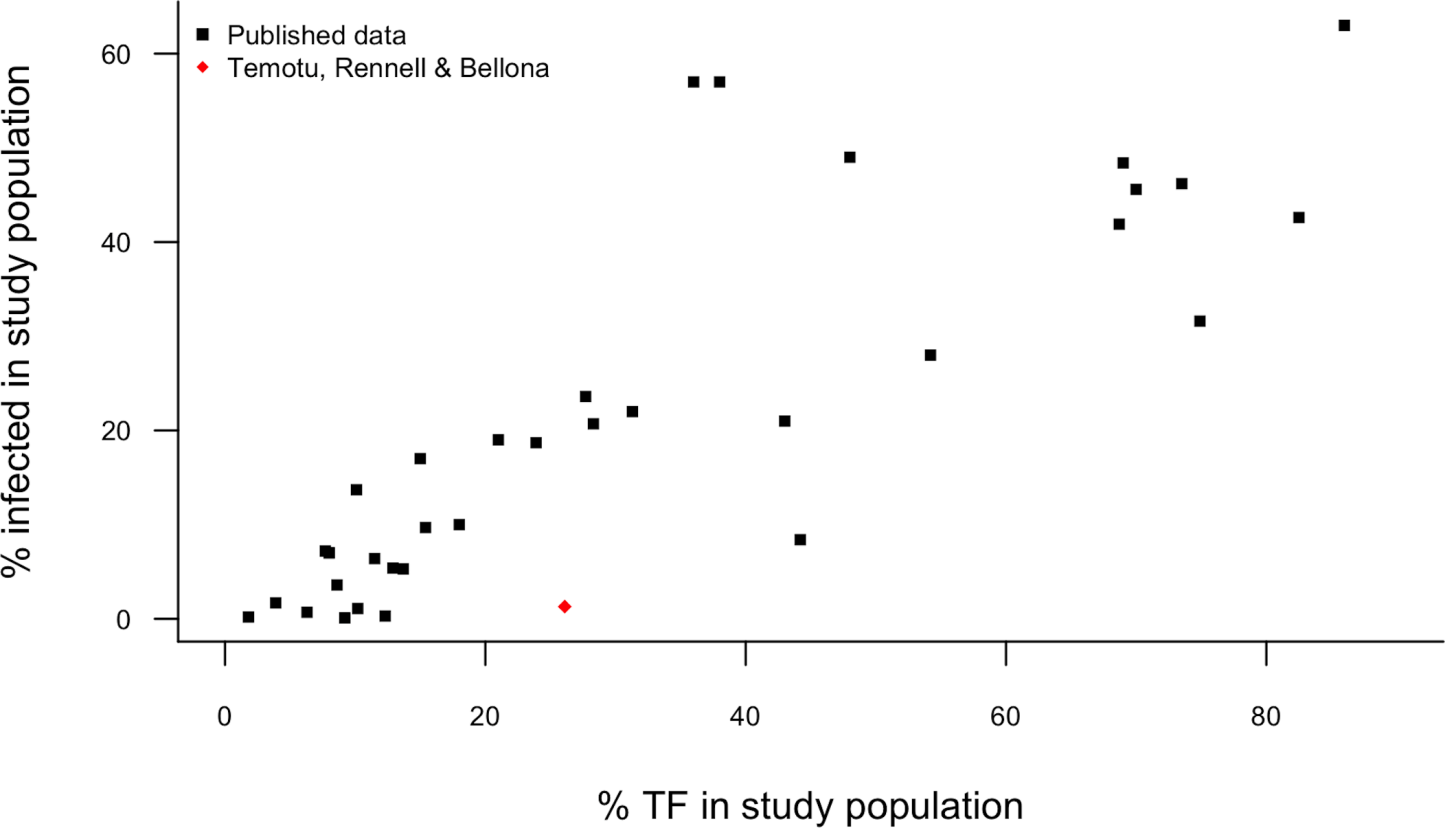
Relationship between the prevalence of conjunctival *C*. *trachomatis* infection (diagnosed by nucleic acid amplification test) and trachomatous inflammation—follicular (TF) in the total 0–9 year-old population or a subset of that group at the district level (n = 35 districts). Correlation coefficient (R) is 0.84.

We identified a number of previous studies have reported concomitant *Ct* infection and active trachoma prevalence estimates [19–21,53–70] in children aged 0–9 years or a subset of that group in 35 districts. At the population level there is a good correlation between the two (R = 0.84) (Fig 6). Our *Ct* infection estimate does not conform to the patterns observed in those other populations, with infection being substantially less prevalent than might be expected given the TF prevalence.

## Discussion

We report an apparently mild trachoma phenotype in which TF is moderately prevalent yet *Ct* infection, TI and TT are rare. The findings reflect those of other studies; over 2300 adults were surveyed in Makira, Isabel and Central provinces yet only 3 cases of TT identified, indicating an unadjusted prevalence among those adults of 0.1% despite 22.2% of the 1–9 years population in the same survey having signs of TF [12]. The TF prevalence in Temotu, Rennell and Bellona is the highest of the populations surveyed during GTMP mapping. Choiseul province had low levels of both TF (6%) and TT (0%). Interestingly, Western Province had more TT cases than Temotu, Rennell and Bellona [71]. Further studies are warranted to determine whether infection prevalence is also higher in that region. The prevalence of TF in these communities qualifies this EU for priority implementation of the A, F and E components of the SAFE strategy (**s**urgery to treat TT, mass **a**ntibiotic distribution to treat infection, promotion of **f**acial cleanliness and **e**nvironmental improvement to reduce transmission [72]), but the trichiasis data suggest the elimination target for TT has already been met. The evidence of this survey suggests that prevalence of TF in children may not be an appropriate marker of disease burden in this setting. Prolonged infection with *Ct*, intense transmission of *Ct*, presence of TI and other markers of inflammation have been associated with progression to TS [3,73–75], the precursor of TT. It is therefore feasible that the low prevalence of TI and *Ct* are related to the paucity of TT in this population.

In Fig 4 we demonstrate that the correlation between reported TF and TT prevalence is weak, suggesting that high TF prevalence is not always indicative of a significant TT burden. This is not an unexpected finding; the signs of TF are transient, being instigated and cleared over the course of weeks or months, whereas TT has a much longer-term onset and is influenced by an accumulation of a lifetime of microbiological and immunological stimuli. Field grading has not previously been standardised across studies and must therefore be compared between studies with caution. TF is used in part for ease and uniformity of field grading rather than specificity for chlamydial infection, and Fig 6 shows that there is a moderate correlation between population *Ct* infection prevalence and population TF prevalence in most districts studied to date. The search criteria for this literature search were relatively lenient; controlling for field grading discrepancy, sample collection methodology, and diagnostic test would likely result in a stronger correlation.

At the individual level, TF and *Ct* prevalence do not always correlate well; for example in a rural Tanzanian community where the TF prevalence was >10%, only 6.1% of those with TF had *Ct* infection and no association was found with clinical signs disease [74]. Follicular inflammation of the conjunctiva can have many different causes, such as viruses, nonchlamydial bacteria, chemical exposure and allergic reactions [76]. While detailed eyelid examination may be able to distinguish these infections phenotypically, the WHO simplified grading system is not sufficiently detailed to do so. *Streptococcus pneumoniae* and *Haemophilus influenzae* have been shown to be significantly associated with TF in the Gambia and Tanzania [74,77]. Numerous other species of the *Staphylococcus*, *Streptococus*, *Moraxella*, *Hameophilus* and *Corynebacteria* genera among others have been cultured from the conjunctivae of children and adults living in trachoma-endemic areas. Although many have not been shown to associate with clinical signs of TF, there are indications that in adults these bacteria can drive inflammation which leads to increased scar tissue deposition [78] or recurrent TT after surgery [79,80]. It is therefore likely that a proportion of all TF cases globally may not be chlamydial in origin; it seems that in this Pacific Island setting, this proportion is high and this is translated into a reduced prevalence of end-stage trachomatous disease. Fig 4 also indicates that this may also be true of other parts of the world, where districts with sufficient TF to qualify for intervention under WHO guidelines do not necessarily have a significant TT burden. Exposure to circulating *Ct* may modulate the immune response at the conjunctiva to increase inflammation in response to otherwise commensal organisms. In turn this could drive the immunopathology that leads to scarring. It is possible that the low prevalence of *Ct* observed in this population is insufficient to drive intense transmission, and children are exposed less frequently than in other trachoma-endemic populations and therefore are not as susceptible to such intense or regular periods of inflammation.

The highest age-specific TF prevalence in this study was in those aged 6 years (Fig 1). The difference between that and other published data, as documented in Fig 5, may imply a different mode or intensity of transmission, or may reflect reduced accumulation of partial immunity. It is not possible to determine the true mechanism without intensive longitudinal study, but this observation supports the case that the epidemiology of TF in our population is atypical.

While the majority of our *Ct*-positive swabs were taken from eyes with active trachoma, we consider the low absolute number of infections insufficient to drive the moderate burden of TF. It is not clear from our cross-sectional study whether a non-chlamydial microbial agent is causing TF, or whether those with TF had suffered a relatively recent *Ct* infection and had persistent inflammatory disease causing the follicular inflammation we observed. The use of ddPCR has not yet become widespread in infectious disease studies. While it is not suitable for all applications, it offers the significant benefit of reference-free quantification of nucleic acids. In the present study, the load of *Ct* in positive samples was substantial, which are thought to be more transmissible than low-load infection. There was also substantially higher infection load in those with TF as compared to those without TF, although this difference was not statistically significant.

Despite recent advances in culture-free sequence methodologies, low-load infections are known to yield poor quality or no sequence data. The technique we used in this study reportedly provides high quality sequence data (20× read depth over at least 95% of the genome) when the input specimen has above ~12,000 and ~98,000 chlamydial genome copies in vaginal swab and urine samples, respectively [25]. A lower load limit for ensuring high quality sequencing in ocular samples is not yet known, but we were encouraged that partial or complete sequence data were yielded from 11/13 *Ct* positive swabs strains that were sequenced. Those where complete genome coverage (>95%) was achieved appeared to be most closely related to ocular serovars, and appeared to be very closely related to each other. The sequence information suggested that, at *trpA* and the wider PZ, the Solomon sequences were closely related to ocular strains, and ocular and urogenital strains were distinct from each other at these loci. The *trpA* open reading frame was truncated in these sequences. This region contains key determinants of *Ct* tissue tropism and further supports the close relationship of these strains to classical ocular references. The small number of sequences available makes it difficult to identify differences potentially related to pathogenicity. Urogenital strains are known to be able to infect conjunctival epithelium [81], and given the high prevalence of sexually transmitted *Ct* infections in the Solomon Islands [82], we may have expected some contamination of the conjunctivae with urogenital chlamydial strains. Our data did indicate urogenital strains were present in several conjunctivae, but the quality of those sequences aligning to urogenital references was uniformly low. It is not possible to determine whether this was because these were urogenital strains that had not established a sufficiently fulminant infection to yield enough material for sequencing, or whether the matches obtained were an artefact of the low sequence coverage. We can say, though, that our next generation sequencing confirmed that strains with high sequence homology to well-defined ocular *Ct* strains are present in the conjunctivae of children in the Solomon Islands.

One limitation of our study is the absence of an alternative explanation for the discrepancy between *Ct* and TF levels. Only samples for which testing passed various quality control steps were included in this paper and our test had been previously validated against an external standard. We therefore do not believe that simple diagnostic failure has significantly influenced our data. Of the four signs of trachoma described in this paper (TF, TI, TT and current infection), three (infection, TI and TT) are present at low levels in this population. Further studies are underway to test for potential alternative pathogens such as *S*. *pneumoniae* and *H*. *influenzae*, and we are investigating longer-term markers of *Ct* infection by screening the population of Temotu, Rennell and Bellona for both trachomatous scarring and antibodies against chlamydial pgp3 antigens. We have not addressed the genetics of the population in this study and while host genetic factors have been shown to associate with an increased risk of scarring [83,84] very little is known about diversity in immune response genes in the Solomon Islands. The limited amounts of immunogenetic typing data that are available indicate that some HLA epitopes associated with increased risk of scarring (e.g. HLA-C2) are moderately prevalent in the Pacific region, while other putative protective alleles (e.g. the HLA-B*08:01~C*03:04 haplotype) are almost absent (data taken from allelefrequencies.net, search March 2016). Investigating this is beyond the scope of this study, but accumulated evidence on the genetics of trachoma indicate that both pathogen and host are sufficiently well adapted to coexistence that a host polymorphism that makes the host entirely refractory, or pathogen variation that completely ameliorates the infectivity and/or pathogenicity of *Ct* seems unlikely. Polymorphisms in key immune genes such as IL-10 and gamma-interferon have been shown to be more frequent in cases with severe trachoma than in normal controls [85], although these were not replicated in genome-wide association screening. It is possible that variation in immune responsiveness may influence the susceptibility of this population to *Ct* infection but a specific immune pathway that is expressed significantly differently between those in whom scarring progresses and those in whom it doesn’t has yet to be identified [75].

In addition to ocular *Ct* infection, we observed signs of both active trachoma and trachomatous conjunctival scarring in this sample indicating trachoma is or has recently been endemic in these islands. However, the prevalence of *Ct* infection appeared to be too low to be the sole explanation for the high burden of TF, while TI and TT were curiously scarce given the substantial amount of TF that was present. Whilst *Ct* may account for some of the TF in this population, we expect that the majority of TF-like disease is either caused by a single as-yet undetermined factor, or by multiple contributory aetiologies. This form of disease, if not unique to the Solomon Islands, might inflate estimates of trachoma burden and could lead to unwarranted mass drug administration in other world populations. In several European settings, there has been a steady increase in incidence and reinfection rates of urogenital *Ct* despite enhanced detection and treatment, which some have hypothesised could be attributed to interruption of the natural acquisition of immunity [86]. We do not have good markers of what constitutes ‘acquired immunity to *Ct’* to measure this, but it is relevant in this context to consider the possibility of negative effects of MDA in addition to the potential positive ones. The findings of this study may have profound impacts on approaches to trachoma programme monitoring in the peri-elimination period.

## Supporting Information

S1 Checklist This manuscript adheres to the “Strengthening the Reporting of Observational Studies in Epidemiology (STROBE)” guidelines [89]. (DOC)

S1 Fig Maximum likelihood phylogram of (**A**) genome and (**B**) plasmid sequences from clinical specimens assembled using *C*. *trachomatis* E/Bour reference. All branches had bootstrap values over 85/100.
(TIFF)

S2 Fig Phylogram illustrating relationship of Solomon Islands sequences to reference sequences at (**A**) ompA, (**B**) trpA and (**C**) PZ regions.
(TIFF)

S1 Table Raw data from this study in accordance with PLoS NTDs editorial guidelines. (TXT)

S2 Table Sequence accession numbers. (DOCX)
