# Correction: Low Prevalence of Conjunctival Infection with *Chlamydia trachomatis* in a Treatment-Naïve Trachoma-Endemic Region of the Solomon Islands

**DOI:** 10.1371/journal.pntd.0005051

**Published:** 2016-10-03

**Authors:** Robert M. R. Butcher, Oliver Sokana, Kelvin Jack, Colin K. Macleod, Michael Marks, Eric Kalae, Leslie Sui, Charles Russell, Helena J. Tutill, Rachel J. Williams, Judith Breuer, Rebecca Willis, Richard T. Le Mesurier, David C. W. Mabey, Anthony W. Solomon, Chrissy h. Roberts

The fifth author's name is spelled incorrectly. The correct name is: Michael Marks. The correct citation is Butcher RMR, Sokana O, Jack K, Macleod CK, Marks M, et al. (2016) Low Prevalence of Conjunctival Infection with Chlamydia trachomatis in a Treatment-Naïve Trachoma-Endemic Region of the Solomon Islands. PLoS Negl Trop Dis 10(9): e0004863. doi: 10.1371/journal.pntd.0004863
