# Supplementary figures and images for: Low Prevalence of Conjunctival Infection with Chlamydia trachomatis in a Treatment-Naïve Trachoma-Endemic Region of the Solomon Islands
Source: PLoS Negl Trop Dis. 2016 Sep 7;10(9):e0004863. doi: 10.1371/journal.pntd.0004863 (PMC5014345; doi:10.1371/journal.pntd.0004863)

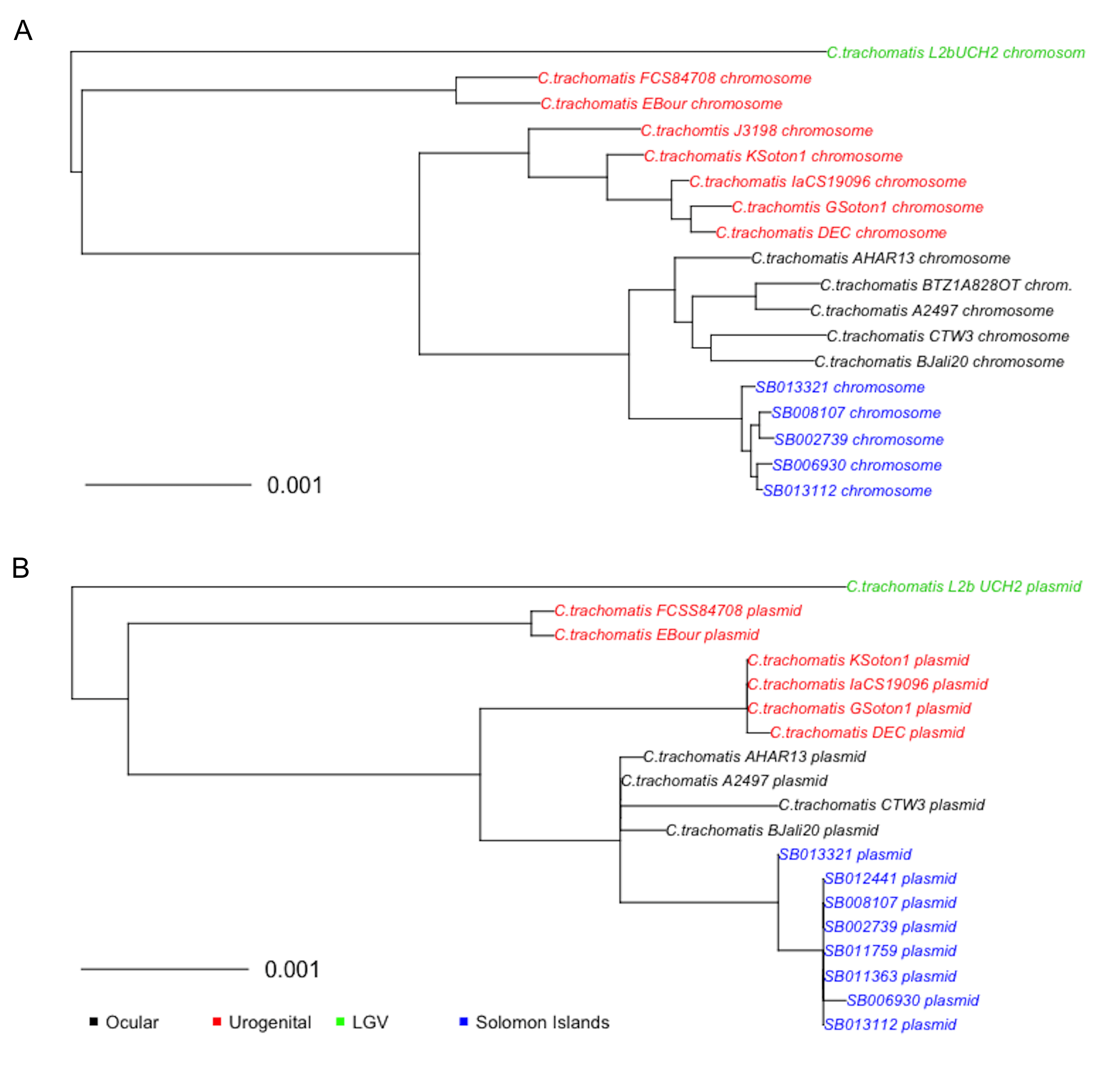

Supplement: S1 Fig — Maximum likelihood phylogram of (A) genome and (B) plasmid sequences from clinical specimens assembled using C. trachomatis E/Bour reference. All branches had bootstrap values over 85/100. (TIFF) [file pntd.0004863.s002.tiff]

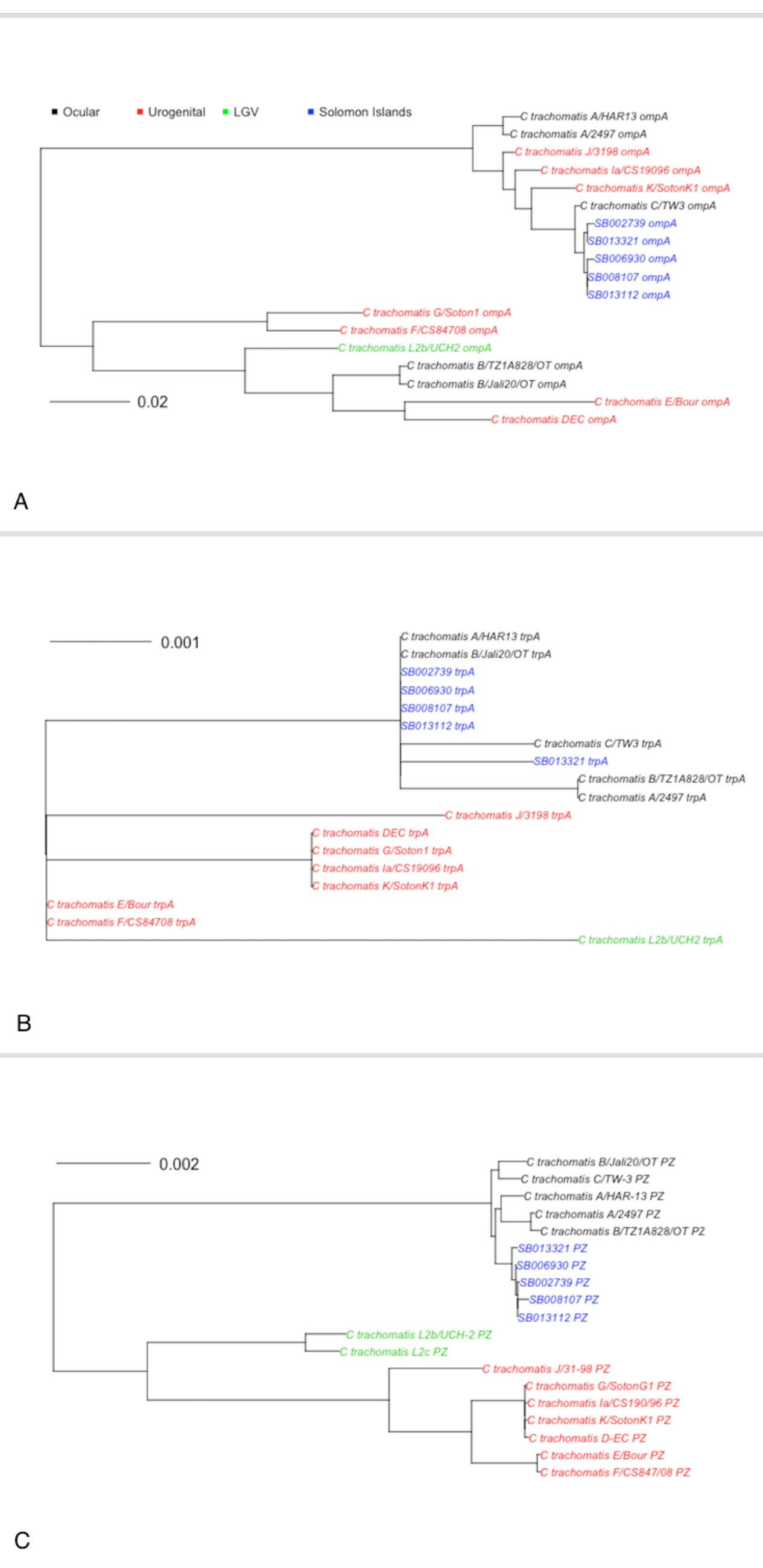

Supplement: S2 Fig — Phylogram illustrating relationship of Solomon Islands sequences to reference sequences at (A) ompA, (B) trpA and (C) PZ regions. (TIFF) [file pntd.0004863.s003.tiff]
