# Supplementary material for: Low Prevalence of Conjunctival Infection with Chlamydia trachomatis in a Treatment-Naïve Trachoma-Endemic Region of the Solomon Islands
Source: PLoS Negl Trop Dis. 2016 Sep 7;10(9):e0004863. doi: 10.1371/journal.pntd.0004863 (PMC5014345; doi:10.1371/journal.pntd.0004863)
Supplement: S2 Table — (DOCX) [file pntd.0004863.s005.docx]

| **Participant ID** | **Chromosome GenBank accession number** | **Plasmid GenBank accession number** |
| --- | --- | --- |
| SB002739 | CP016418 | CP016419 |
| SB006930 | CP016420 | CP016421 |
| SB008107 | CP016422 | CP016423 |
| SB013112 | CP016424 | CP016425 |
| SB013321 | CP016426 | CP016427 |

Butcher *et al.*, Low prevalence of conjunctival infection with Chlamydia trachomatis in a treatment-naïve trachoma-endemic region of the Solomon Islands. *PLOS NTDs.*

**Supplementary table 2.** Sequence Accession numbers
